# Transcutaneous vagus nerve stimulation in breast cancer: a neuroimmune model to improve quality of life

**DOI:** 10.3389/fonc.2026.1731999

**Published:** 2026-02-04

**Authors:** Melissa Do, William J. Tyler

**Affiliations:** 1 Department of Biomedical Engineering, University of Alabama at Birmingham, School of Engineering, Birmingham, AL, United States; 2 Center for Neuroengineering and Brain Computer Interfaces, Heersink UAB School of Medicine, Birmingham, AL, United States

**Keywords:** anxiety, autonomic regulation, breast cancer, fatigue, inflammation, insomnia, quality of life, vagus nerve stimulation

## Abstract

Breast cancer care has shifted beyond remission toward optimizing long-term physiological, emotional, and functional recovery. Many survivors continue, however, to experience persistent symptom clusters, such as insomnia, fatigue, anxiety, pain, depression, and cognitive impairment. These poor quality of life outcomes reflect underlying dysregulation of autonomic, neuroendocrine, and immune systems. Autonomic imbalance characterized by vagal withdrawal and sympathetic hyperactivation is linked to increased inflammatory load, impaired stress regulation, disrupted sleep, and poorer survival outcomes. Given the role of the vagus nerve in coordinating brain–body signaling and immune modulation, transcutaneous vagus nerve stimulation (tVNS) has emerged as a promising intervention to restore autonomic balance and attenuate psychophysiological burdens. Evidence suggests that tVNS modulates locus coeruleus–norepinephrine activity, regulates arousal and sleep, reduces fatigue and anxiety, enhances cognitive function, and activates the cholinergic anti-inflammatory pathways. Supported by mechanistic and clinical evidence, we propose tVNS as a precision-guided bioelectronic strategy for improving survivorship outcomes in breast cancer.

## Introduction

Breast cancer is one of the most frequently diagnosed cancers globally, accounting for more than 2 million new cases a year (1). While treatment therapies have advanced significantly, disease and treatment consequences remain poorly addressed. Patients with breast cancer, beginning at diagnosis and into active treatment and survivorship, often endure persistent physiological and psychological symptoms such as insomnia, fatigue, anxiety, depression, pain, inflammation, and cognitive dysfunction (2–6). As a result, approximately 42%–45% of patients with breast cancer suffer from emotional distress, which has been formally recognized as the “sixth” vital sign because of its significant impact of physical and psychological health (7, 8). These symptoms rarely occur in isolation but instead form interrelated clusters, often exacerbating one another and are associated with poorer treatment outcomes, elevated inflammatory markers, reduced treatment adherence, slower recovery, decreased overall health, and potentially reduced survival (9–11).

Breast cancer treatments, including surgery, chemotherapy, radiation therapy, and immunotherapy, further disrupt psychological resilience and distress, as well as the incidence of pain and inflammation (12). The most common treatment for breast cancer is surgery. Aside from fatigue, pain, and reduced mobility associated with surgery, changes in body image and self-perception following procedures like mastectomy can cause stress and depression (13, 14). Studies have shown that mastectomy is associated with increased severity of depression and anxiety, especially when compounded by the compromise of self-image (15). Similarly, the prevalence of depression was shown to increase in patients with breast cancer after chemotherapy (16, 17), radiation therapy (18, 19), and immunotherapy (14, 20). Symptoms can often persist after the cessation of treatment.

Women are often prescribed adjuvant hormone therapy for 10 years following primary breast cancer treatment to reduce cancer recurrence and morality (21). Hormone therapy is extremely effective in inhibiting hormone production or by interfering with hormone receptor signals to prevent tumor growth when taken as prescribed (22). However, hormone therapy was reported to increase the risk or worsen depressive symptoms. The most common and severe side effects reported from hormone therapy treatment include sleep disturbances, fatigue, and depression, which have been associated with higher rates of depression (23). These adverse effects not only impair quality of life (QoL) but also serve as predictors of poor treatment adherence (24).

Despite its high prevalence and impact in patients with cancer, effective and systematic treatment interventions remain limited. Supportive and survivorship care serves to address these comorbidities, and while guidelines are well established, many patients in the United States report suboptimal results, leaving patients with cancer with persistent symptoms, unmet needs, poor coordinated treatment plans, and non-accessible comprehensive care (25–27). Primary providers often address many of these symptoms with pharmaceutical interventions such as benzodiazepines, non-benzodiazepine hypnotics, antidepressants, opioids, and non-pharmaceutical interventions such as cognitive behavioral therapy (CBT) (28–30). Pharmaceutical interventions, while effective, are often associated with risks of severe adverse effects and drug abuse or dependence (28, 31). Studies have shown that CBT is effective and long-lasting for a variety of mental and mood disorders. Despite this, up to 26% of patients prematurely drop from therapy (32). In addition, access to CBT is limited and underutilized (33). Although increasing awareness and substantial diagnosis and therapeutic advances have been made to address breast cancer, there remain unmet needs for addressing symptom clusters in patients with breast cancer.

Emerging evidence indicates that these symptom clusters reflect shared disruptions in autonomic and neuroimmune regulation, marked by sympathetic overactivation, vagal withdrawal, and heightened inflammatory activity (2, 34, 35). Reduced heart rate variability (HRV), a validated biomarker of autonomic dysregulation and vagal tone, has been associated with increased inflammatory burden, impaired emotional regulation, decreased stress resilience, cognitive dysfunction, and reduced survival outcomes (36–39). Indeed, low HRV is frequently observed in patients with breast cancer and correlates with elevated circulating cytokines such as interleukin-6 (IL-6) and tumor necrosis factor-alpha (TNF-α), increased sympathetic drive, poorer QoL, and heightened mortality risk (37, 39). Collectively, this evidence positions autonomic dysregulation and inflammation as central mechanisms underpinning disease outcome and adverse symptom burden.

**Figure 1:**
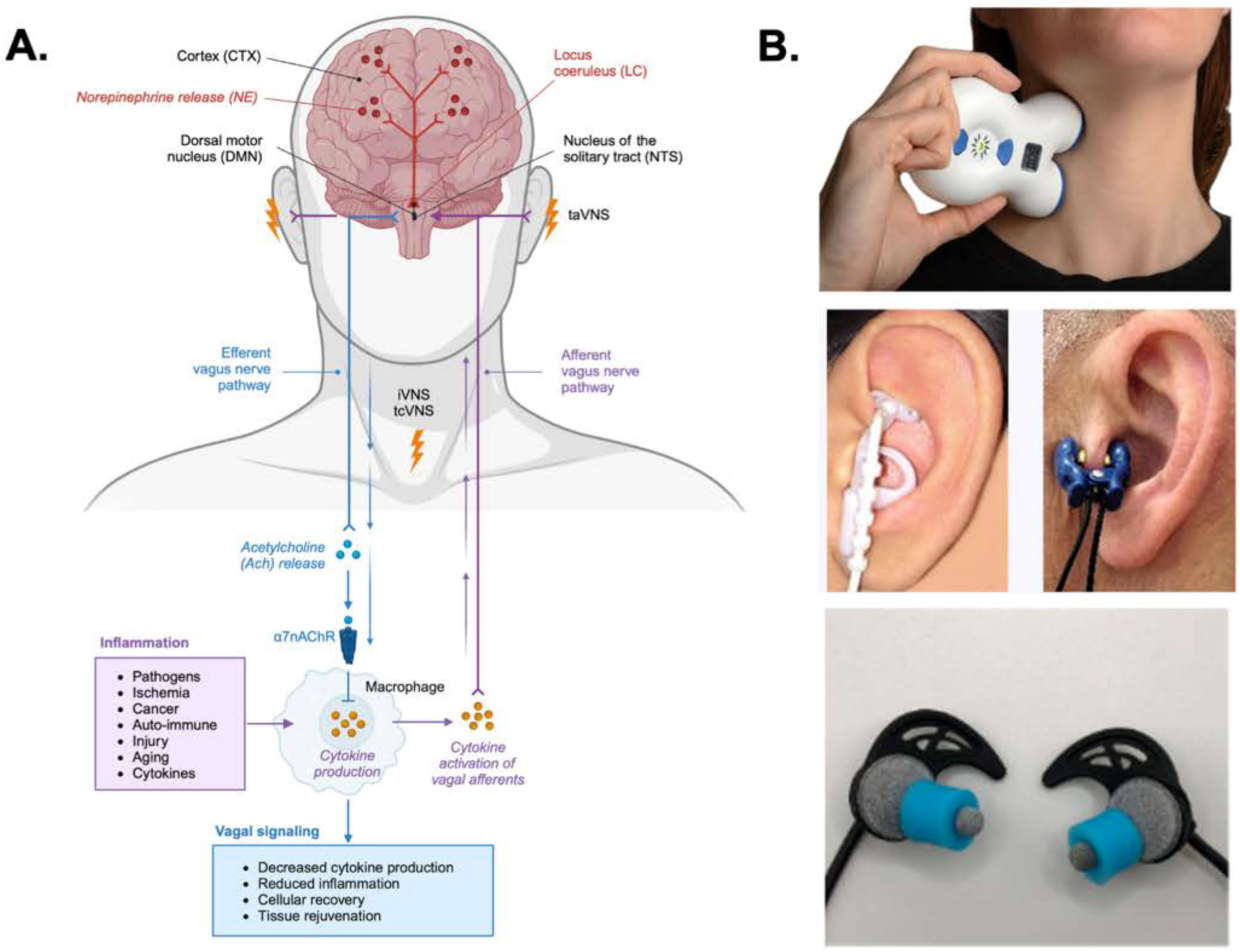
Afferent and efferent pathways of transcutaneous vagus nerve stimulation (tVNS) and their neuroimmune effects. **(A)** Schematic representation of auricular (taVNS) and cervical (tcVNS) stimulation targeting vagal afferents projecting to the nucleus tractus solitarius (NTS), with downstream modulation of the locus coeruleus (LC), dorsal motor nucleus (DMN), and norepinephrine (NE) release within central arousal and autonomic regulatory circuits. Efferent vagal signaling engages the cholinergic anti-inflammatory pathway, whereby acetylcholine (ACh) release activates α7 nicotinic acetylcholine receptors (α7nAChR) on immune cells, reducing cytokine production and inflammation and promoting cellular recovery and tissue restoration. ACh, acetylcholine; DMN, dorsal motor nucleus; LC, locus coeruleus; NE, norepinephrine; NTS, nucleus tractus solitarius; taVNS, transcutaneous auricular vagus nerve stimulation; tcVNS, transcutaneous cervical vagus nerve stimulation; α7nAChR, α7 nicotinic acetylcholine receptor. **(B)** Common methods of transcutaneous cervical vagus nerve stimulation (tcVNS) and taVNS are illustrated.

The vagus nerve sits at the center of psychophysiological regulation and may serve as a key mechanistic hub for therapeutic intervention in the QoL and survivorship in patients with breast cancer. As the primary component of the parasympathetic nervous system, the vagus nerve (cranial nerve X) provides extensive afferent and efferent innervation between the brain and visceral organs, including the heart, lungs, gastrointestinal tract, and immune system (**Figure 1A**). Afferent vagal inputs to the nucleus tractus solitarius (NTS) regulate the locus coeruleus–norepinephrine (LC-NE) system, modulate hypothalamic–pituitary–adrenal (HPA) axis reactivity, influence glucocorticoid sensitivity, and activate the cholinergic anti-inflammatory pathway (CAIP) (40–42). Through these networks, the vagus nerve governs arousal regulation, cardiovascular activity, stress resilience, emotional processing, cognitive function, immune tone, and systematic inflammatory signaling, all of which are domains disrupted in patients with breast cancer (**Figure 1A**). Furthermore, reduced vagal tone has been associated with increased risk of recurrence and poorer overall survival in breast cancer, suggesting that persistent neurophysiological distress may contribute to poor recovery and tumor-permissive biological states (37, 39, 43, 44).

Modern vagus nerve stimulation (VNS) began with the development of implantable devices in the late 20th century, with the first device approved for epilepsy in 1988 (45, 46). The success of invasive VNS in epilepsy management led researchers to explore its therapeutic potential across a range of conditions, and nearly 125 years after VNS was first described, non-invasive VNS emerged as a more accessible, lower-cost alternative (47). Today, transcutaneous vagus nerve stimulation (tVNS), embodied as transcutaneous auricular VNS (taVNS) targeting the auricular branch of the vagus nerve (ABVN) or transcutaneous cervical VNS (tcVNS) stimulating the cervical branch, has gained widespread attention for demonstrating safe and effective treatment in numerous conditions (**Figure 1B**). These tVNS approaches have been shown to treat depression, insomnia, migraine, chronic pain, posttraumatic stress disorder (PTSD), post-stroke rehabilitation, anxiety, epilepsy, and cognitive impairment (48–52). It is well established that VNS works, in part, by modulating the activity of the locus coeruleus (LC), a key noradrenergic nucleus within the ascending reticular activating system of the brainstem (53–56). This modulation drives cortical activation and regulates transitions across arousal states. Through activation of NTS-mediated networks in humans, non-invasive stimulation of vagal sites has been shown to influence LC firing patterns and alter norepinephrine release throughout the brain, with downstream effects on arousal, attention, neural plasticity, emotional regulation, and autonomic balance (45, 52, 57–60).

Our recent open-label pilot study in women with breast cancer-related insomnia showed that nightly, bilateral taVNS is safe, well tolerated, and capable of improving sleep and psychophysiological outcomes, reinforcing its translational relevance for supportive and survivorship care (61). Early clinical studies across diverse populations further demonstrate the capacity for tVNS to improve symptoms in insomnia, anxiety, depression, pain, fatigue, and cognitive dysfunction, further supporting its potential as a psychophysiological regulator (49–51, 62). Taken together, tVNS presents a promising method for targeting the interconnected symptom clusters in patients with breast cancer through restoration of vagal tone, modulation of neuroimmune circuitry, and regulation of arousal pathways. Therefore, we propose a model that centers on the potential of tVNS to mitigate insomnia, fatigue, affective dysregulation, pain, and cognitive impairment by addressing autonomic and inflammatory dysregulation that impact the QoL and survivorship in patients with breast cancer. By integrating current evidence from neuroscience, bioelectronic medicine, and psychoneuroimmunology, we present a conceptual and empirically grounded framework for incorporating tVNS into breast cancer treatment models to support patient disease recovery, survival, and improve physical, emotional, and mental distress.

## Symptom clusters in breast cancer survivorship: a neuroimmune–autonomic perspective

### Conceptualizing symptom clusters

Breast cancer and standard oncologic treatments can cause multisystem disruption across essential homeostatic regulatory systems, including those involved in immune activity, neuroendocrine signaling, and autonomic balance. These disruptions can impair treatment compliance, accelerate disease progression, and worsen clinical outcomes (2, 3, 63, 64). Over time, this dysregulation contributes to chronic fatigue, heightened inflammatory activity, and reduced physiological resilience in patients and survivors (2, 64).

**Figure 2:**
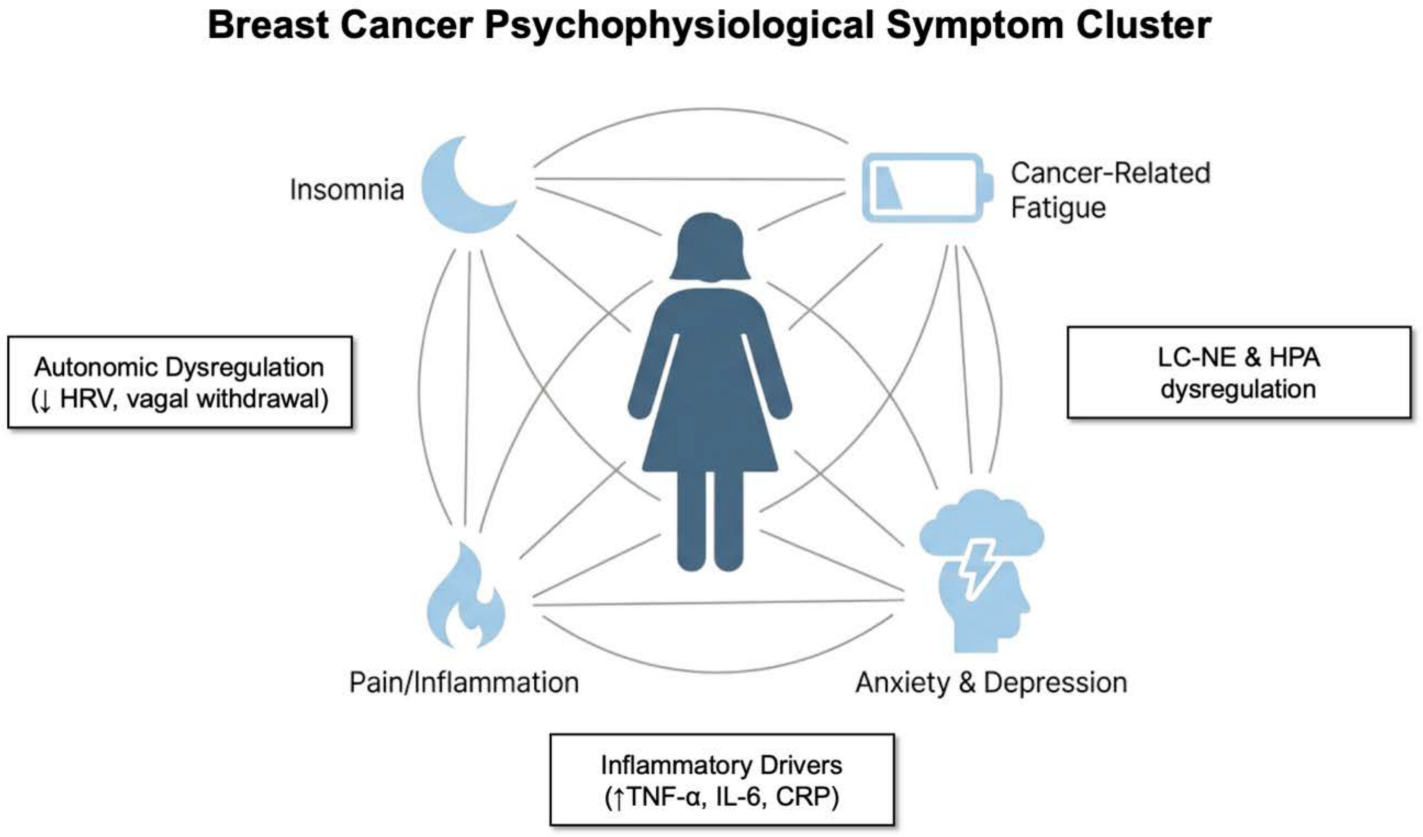
Psychophysiological symptom clusters in breast cancer and their bidirectional association with autonomic and inflammatory dysregulation. As the illustration depicts, fatigue, insomnia, pain, and anxiety/depression cluster as interdependent and mutually reinforcing cancer-related symptoms that exacerbate one another through bidirectional feedback loops. These symptom interactions emerge within a context of autonomic imbalance (e.g., reduced HRV, vagal withdrawal, and sympathetic hyperarousal), heightened inflammatory activity (e.g., elevated TNF-α, IL-6, and CRP), and dysregulation of central stress circuits (e.g., LC–NE and HPA axis activation), which collectively perpetuate chronic psychophysiological burden and reduced resilience. HRV, heart rate variability; LC–NE, locus coeruleus–norepinephrine; HPA, hypothalamic–pituitary–adrenal axis; CRP, C-reactive protein; TNF-α, tumor necrosis factor-alpha; IL-6, interleukin-6.

Cancer-related symptoms such as insomnia, fatigue, depression, anxiety, and pain are highly prevalent in breast cancer survivors and frequently co-occur rather than presenting as isolated complaints. The concept of symptom clusters demonstrates interdependent phenomena where individual symptoms often amplify one another, such that the presence of one increases the incidence and severity of others (**Figure 2**), ultimately compounding overall symptom burden (2, 64). Although these symptoms may present differently and emerge at various stages along the disease trajectory, they often share a common etiology that may be amenable to targeted intervention, thus gaining increased recognition in oncology research (65, 66).

Meta-analytical syntheses highlight that fatigue–sleep disturbance and psychological clusters, encompassing anxiety, depression, nervousness, irritability, sadness, and worry, are among the most consistently reported and burdensome symptoms in breast cancer populations (**Figure 2**). Additional empirical and meta-analytic evidence further indicates that fatigue–sleep disturbance and psychological symptom clusters (e.g., anxiety, depression, irritability, sadness, and worry) are the most frequently observed in patients with breast cancer (2, 5, 64, 67, 68). These symptom constellations form dynamic, interdependent networks rather than independent outcomes and suggest the presence of shared physiological drivers that sustain chronic distress. Understanding symptom clusters can therefore guide the development of effective and integrated care strategies in women with breast cancer. Accordingly, research has increasingly focused on identifying symptom groups to develop strategies that can address multiple symptoms simultaneously (2, 3, 63, 64). This approach has the potential to optimize clinical efficiency, reduce healthcare burden, and improve QoL for patients by enabling more comprehensive and mechanism-informed symptom management.

### Clinical burdens

**Figure 3:**
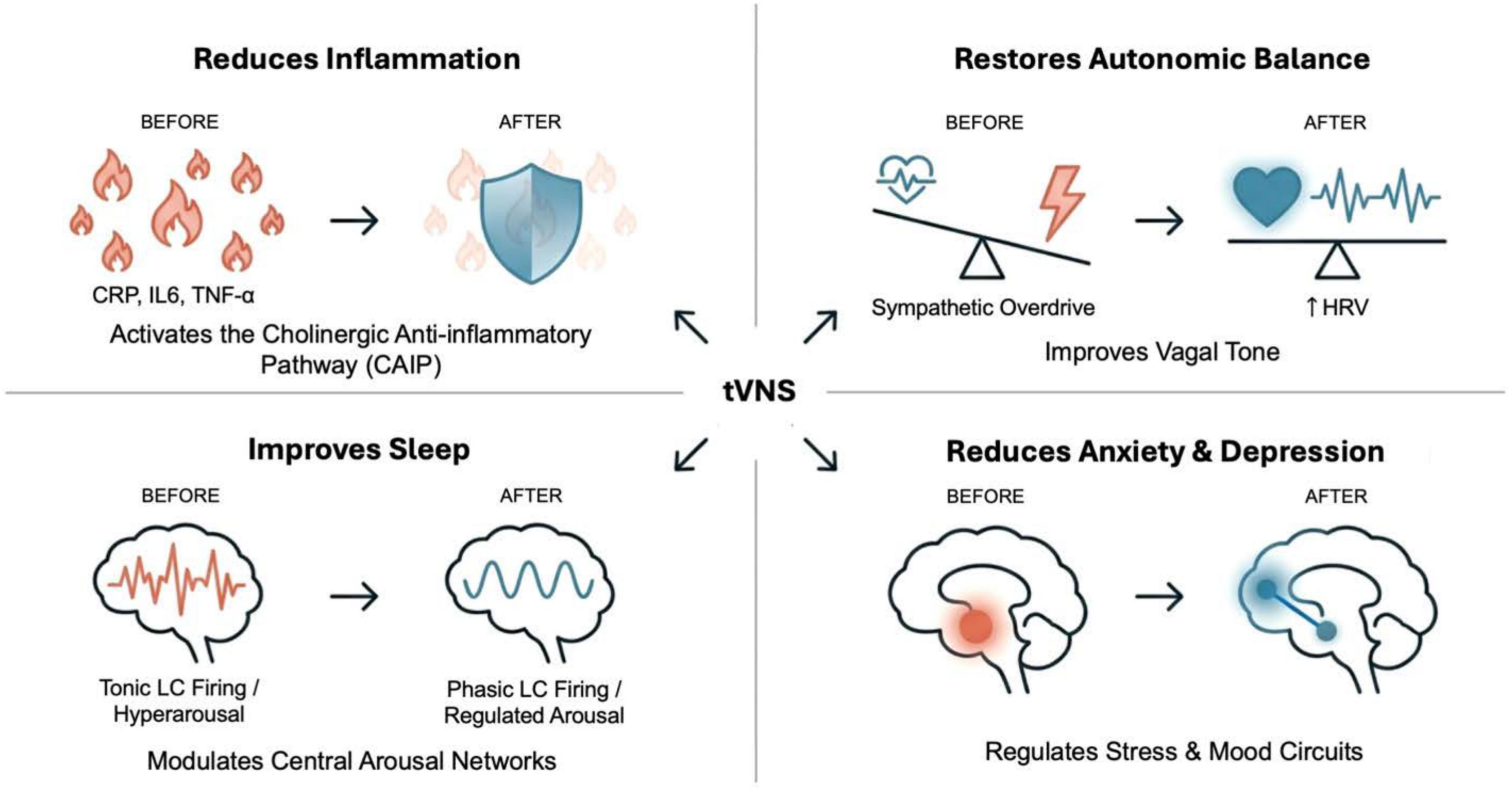
Effects of transcutaneous vagus nerve stimulation on neuroimmune and psychophysiological dysregulation in breast cancer survivors. Prior to intervention, autonomic imbalance, noradrenergic activity, hyperarousal, inflammatory load, and disrupted sleep reinforce a self-perpetuating cycle of fatigue, anxiety, pain, and cognitive effort. As illustrated, transcutaneous vagus nerve stimulation (tVNS) can disrupt these loops by restoring phasic locus coeruleus (LC) activity, enhancing parasympathetic tone, reducing inflammatory cytokine burdens, and improving sleep architecture, thereby contributing to reductions in fatigue, anxiety, depression, and cognitive strain to promote psychophysiological recovery. CAIP, cholinergic anti-inflammatory pathway; CRP, C-reactive protein; IL-6, interleukin 6; TNF-α, tumor necrosis factor-alpha; HRV, heart rate variability.

Patients with breast cancer who present with high symptom clusters often report increased psychological distress, greater functional impairments, significantly lower rates of treatment adherence, and reduced health-related QoL compared to those with lower symptom clusters (11, 69, 70). Rather than emerging as distinct consequences of cancer treatment, these symptoms are increasingly recognized as manifestations of a shared underlying physiological disruption (**Figure 3**). Synergistic effects within clusters lead to cumulative cognitive-emotional load, decreased resilience, impaired daily functioning, and increased healthcare utilization (2, 71). In breast cancer survivors, sleep disruption and fatigue often precede and exacerbate affective dysregulation, which in turn amplifies pain perception and cognitive impairment (19, 72). These symptom clusters have been linked not only to diminished QoL but also to poorer overall survival and higher recurrence risk, underscoring the need for targeted interventions capable of addressing symptom burden (23, 73, 74).

### Inflammation across clusters

Inflammatory processes appear to play a central role in the symptom cluster of sleep difficulties, fatigue, and depression (14, 75–77). Preclinical and clinical evidence supports pro-inflammatory cytokine release as a key mechanism in the development and persistence of cancer-related symptoms (66, 78, 79). Elevation of these cytokines has been associated with fatigue, depressive and anxiety symptoms, pain, and cognitive impairment in breast cancer populations (72, 80, 81), as well as with tumor progression, metastasis, and poorer prognosis (82, 83). Furthermore, pro-inflammatory cytokines contribute to sleep fragmentation and prolonged sleep latency, promote energy depletion via mitochondrial dysregulation, enhance nociceptive signaling, and trigger “sickness behavior” phenotypes characterized by fatigue, negative mood, reduced motivation, and cognitive slowing (81, 84). Chronic inflammation is associated with impaired executive function, reduced attentional control, and deficits in working memory (85). This multifaceted cytokine involvement supports the hypothesis that inflammatory amplification is not incidental but functions as a mechanistic convergence point that underlies symptom cluster expression. Given this well-established link between cancer and inflammation, targeting inflammatory modulation presents a compelling pathway for mitigating symptom burden in patients with breast cancer.

### Autonomic dysregulation and reduced vagal tone across clusters

Converging evidence suggests that autonomic dysregulation, characterized by reduced vagal tone and increased sympathetic activation, not only influences tumor biology and immune function but also emerges as an underlying mechanism that permits symptom clustering in patients with breast cancer (2, 35, 37, 39, 86). Decreased vagal activity disrupts key inhibitory mechanisms over sympathetic arousal and inflammatory activation via the CAIP (**Figure 3**). This results in increased norepinephrine signaling and β-adrenergic stimulation of immune cells, promoting the transcription of pro-inflammatory cytokines including IL-6, TNF-α, and C-reactive protein (CRP) (86, 87), which are associated with fatigue, sleep disruption, mood disorders, pain, and cognitive impairment (81, 88). Indeed, persistent low vagal tone has been identified as a predictor of higher inflammatory load, greater fatigue, poorer sleep quality, and increased emotional distress in cancer populations, implying that autonomic imbalance plays a fundamental role in long-term psychophysiological and QoL outcomes (72, 73).

HRV-based studies demonstrate that greater vagal withdrawal correlates with increased cluster intensity across symptom cluster networks (2, 37). This autonomic profile is recognized in patients with breast cancer, where higher HRV, indexing increased vagal tone, predicts decreased tumor burden, treatment adherence, and longer survival (37, 38). Breast cancer survivors with insomnia, fatigue, depression, and pain consistently demonstrate lower HRV compared to healthy controls, indicating diminished parasympathetic influence (34, 39, 87, 89–93). Within this framework, symptom clusters are reflections of conceptualized chronic autonomic and inflammatory dysregulation. This model supports a therapeutic strategy aimed at restoring vagal tone and rebalancing autonomic function to simultaneously reduce systemic inflammation and improve multi-symptom burden.

It is important to note that parasympathetic nervous system stimulation (via the vagus nerve) has been hypothesized to lead to decreases in the production of pro-inflammatory cytokines through the release of acetylcholine (86). In general, activation of the sympathetic branch of the ANS leads to increased inflammation and activation of the parasympathetic branch leads to decreased inflammation (94). Indeed, studies have documented cross-sectional associations between higher parasympathetic activity, as indexed by HRV, and lower levels of inflammation (95–97). Taken together, these observations support an autonomic nervous system (ANS) framework as a unifying mechanism in breast cancer-related symptoms. This framework will be used as a recurring interpretive lens throughout subsequent sections to contextualize the autonomic, inflammatory, and neurocognitive mechanisms underlying individual symptom clusters.

### A dysfunctional psycho-neuroimmune loop

These symptom clusters appear to be sustained and permitted by recurrent feedback loops involving autonomic dysregulation, inflammatory signaling, affective processing, and cognitive appraisal (**Figure 2**) (98). For example, psychological distress related to breast cancer frequently emerge or intensify during cancer treatment and may persist long after its treatment ends. Chronic behavioral and psychological comorbidities, combined with the side effects of primary and adjuvant treatments, markedly increase the risk of insomnia, while sleep disturbances further exacerbate this distress and increase inflammatory cytokine expression (99). In turn, fatigue and anxiety are heightened. Fatigue reduces activity levels, which leads to increased pain sensitivity and negative affect. Negative affect further disrupts sleep and cognitive processing, perpetuating the cycle (67, 100).

Breast cancer is also associated with dysfunction across multiple biological systems, including immune, endocrine, and neurological pathways, which further contribute to the onset and persistence of these symptom clusters (14, 77). As previously established, inflammatory processes play a central role in the symptom cluster of sleep difficulties, fatigue, and depression, while poor sleep has been consistently associated with elevated inflammatory markers (4). Studies have shown that insomnia in cancer populations is strongly associated with elevated rates of depression and anxiety (18), as well as impaired immune function. Indeed, depression in breast cancer survivors can emerge through several etiological factors, including psychological stressors, physiological dysfunction, and treatment side effects. Diagnosis is life-altering and often gives rise to significant psychological and emotional distress, including a sense of crisis, uncertainty about prognosis, financial burdens, fear of recurrence, and disruption to a patient’s sense of normalcy (101). To reiterate, these symptoms may present differently and emerge at various stages along the disease trajectory but often co-exist and share etiological underpinnings. Studies report that as many as 90% of patients diagnosed with depression complain of sleep disturbances and poor quality of sleep (102), and individuals with insomnia are over four times more likely to develop depression (103). Risks and severity of mental health increases with disease severity and the presence of symptoms such as pain, fatigue, and sleep disturbances (23). Evidence of comorbidity and reciprocal interactions reinforce a chronic psychophysiological stress state that is resilient to single-symptom therapeutic approaches.

### Rationale for vagal intervention

Breast cancer-related symptom cluster burden may therefore be conceptualized as a state of altered neuroimmune homeostasis, where dysregulation of ANS function interacts with persistent immune activation to maintain symptom clusters (**Figure 2**, **Table 1**). Studies show that higher inflammatory burden and lower vagal tone jointly predict worse fatigue, sleep efficiency, depressive affect, and cognitive complaints in survivors (35, 73). This has led to growing interest in neuromodulatory interventions targeting autonomic restoration, particularly therapies that enhance parasympathetic (vagal) activity, to interrupt inflammatory signaling and ameliorate symptom clusters (86, 104). Such approaches offer a mechanistic alternative to symptom-specific treatments by addressing synergy and overlap in neuroimmune–autonomic circuits (**Figure 3**). Non-invasive VNS is uniquely positioned to engage afferent vagal circuits, shift autonomic balance, reduce inflammatory signaling through CAIP activation, and recalibrate neural systems involved in arousal, emotional regulation, pain processing, and cognitive function [(**Figure 3**, 58, 104–106)]. Therefore, tVNS represents a theoretically grounded, neuromodulatory approach to treating psychophysiological burden in patients with breast cancer. This sets the stage for a mechanistic exploration of how tVNS may intersect with each symptom domain.

**Table 1:** Mechanisms and biomarkers of burdensome symptom clusters in breast cancer.

Symptom cluster	Neuroimmune mechanisms	Psychophysiological (arousal) mechanisms	Key biomarkers
Insomnia and sleep disturbance (hyperarousal)	Inflammatory activation disrupts sleep regulatory circuits. Chronic stress and reduced vagal input promote tonic locus coeruleus–norepinephrine (LC–NE) overactivation.	Physiological hyperarousal. Sympathetic dominance and reduced vagal tone. Sustained activation of wake-promoting systems.	Reduced heart rate variability (HRV) indexing weakened vagal tone. Elevated interleukin-6 (IL-6), tumor necrosis factor-alpha (TNF-α), and C-reactive protein (CRP).
Anxiety and depression (affective dysregulation)	Elevated inflammatory tone. HPA axis dysregulation (e.g., elevated catecholamines and cortisol) is triggered by chronic stress. High chronic cytokine levels increase depressive and anxiety symptoms.	Autonomic imbalance and sympathetic dominance. Vagal withdrawal limits the capacity for emotional regulation. Chronic sympathetic activation and dysregulated LC-NE signaling worsen anxiety.	Lower HRV (associated with diminished emotional regulation). Elevated IL-6, TNF-α, and CRP. Dysregulated cortisol rhythms.
Cancer-related fatigue (CRF)	High inflammation load driven by autonomic dysregulation. Proinflammatory cytokines (e.g., CRP, IL-6, and TNF-α) correlate with greater fatigue severity.	Reduced vagal tone and shift toward sympathetic dominance. Weakened inhibitory control over inflammatory pathways due to disrupted cholinergic anti-inflammatory pathway (CAIP) reflex.	Lower high-frequency heart rate variability (HF-HRV). Elevated IL-6 and TNF-α. Flattened diurnal cortisol rhythms.
Pain and nociceptive amplification	Inflammatory processes sustain pain by causing cytokine-driven nociceptive sensitization. Cytokines such as IL-1, IL-6, and TNF-α contribute to central sensitization and increased nociceptor excitability.	Autonomic dysregulation. Increased sympathetic tone enhances nociceptor sensitization. Impaired vagal tone reduces inhibitory descending pain modulation.	Reduced HRV (correlated with heightened pain intensity). Elevated IL-6, TNF-α, and IL-1β.
Cognitive impairment	Chronic inflammation is associated with impaired executive function and reduced attentional control. Pro-inflammatory cytokines trigger brain fog or cognitive slowing.	Chronic distress and low parasympathetic tone promote tonic LC-NE overactivation (hyperarousal), which contributes to cognitive impairment.	Decreased HRV. Diminished pupillary reactivity or pupillary dilation (as scalable proxies of tonic LC firing and cognitive effort).

## Psychophysiological clusters: inflammatory and autonomic mechanisms

### Insomnia and hyperarousal: cytokine activation, dysregulated arousal, and sympathetic dominance

Insomnia is one of the most prevalent and debilitating symptoms reported by patients with breast cancer, often persisting for years following treatment (107). Patients with breast cancer report the highest rates of insomnia and fatigue compared to other cancer populations, with prevalence estimates ranging from 38% to 70% (12, 108). Sleep deprivation and poor sleep quality is a critical concern for breast care clinicians because it is associated with poorer treatment outcomes, slower recovery, increasing risk of disease progression, decreased overall health, and potentially reduced survival (18, 63, 109). A case–control study that examined sleep quality and duration in women with advanced sleep cancer found that patients with breast cancer who experience greater sleep disturbances show higher mortality rates compared to those who report better sleep quality (110). Moreover, poor sleep quality in patients with breast cancer are associated with increased stress and depressive symptoms, decreased immune function, impaired cognitive functioning, reduced memory consolidation, and deteriorated physiological health (111–114). These symptoms can significantly compromise mental, emotional, and physical functioning, potentially leading to negative personal, professional, and social consequences and reduced QoL (Colten, 2006; Ho, 2015). This is particularly concerning in vulnerable populations who face cumulative health risks and multifaceted disorders (63, 109). The systemic nature of these effects suggests that insomnia in patients with breast cancer reflects deeper physiological dysregulation, particularly involving autonomic imbalance and inflammatory activation (**Table 1**).

Sleep disturbance is closely linked to hyperarousal, inflammatory activation, and disrupted autonomic regulation (2, 4). Breast cancer survivors exhibiting insomnia often show reduced HRV, indicating diminished vagal tone and heightened sympathetic dominance, signatures consistent with physiological hyperarousal (**Table 1** (39, 91);. Inflammatory mediators such as IL-6, TNF-α, and CRP further exacerbate arousal, disrupt sleep regulatory circuits, and reinforce sympathetic dominance (80, 115, 116). These cytokines can interfere with sleep–wake modulatory pathways by promoting glial activation and altering neuromodulatory signaling in hypothalamic and brainstem pathways involved in sleep consolidation and circadian regulation (117, 118). Indeed, elevated levels of pro-inflammatory cytokines are associated with increased sleep latency, higher wake after sleep onset (WASO), and reduced slow-wave sleep (80, 116, 119, 120).

Autonomic dysregulation further contributes to insomnia pathophysiology. The ANS is a control system for homeostatic functions and visceral adjustments that are involved in secretory activities that mediate sleep (121). Neuroanatomical studies demonstrate that sleep–wake regulatory nuclei are closely integrated with regions governing autonomic function, suggesting that ANS is integrally related to sleep physiology (122). In addition, sleep disorders are often reported as manifestations of symptoms of autonomic impairment (57). Indeed, extensive literature supports the role of the ANS in modulating cognitive processes across sleep and arousal biology (57, 120, 122, 123). When this regulatory balance is disrupted, sleep disturbance can trigger sympathetic overactivation and reduce vagal tone, initiating a state of physiological hyperarousal and autonomic imbalance (57). Even transient disruptions in sleep can increase sympathetic drive and inflammatory signaling, perpetuating an autonomic–inflammatory feedback loop that perpetuates hyperarousal and impairs autonomic function (120). To the extent that sleep architecture exerts direct effects on autonomic regulation, autonomic dysfunction may conversely induce sleep disturbances, regardless of the underlying etiology.

Insomnia is increasingly conceptualized as a disorder of hyperarousal, characterized by elevated tonic LC-NE firing, sympathetic dominance, reduced vagal tone, and persistent activation of wake-promoting systems that resist transition into sleep (119, 124). This imbalance drives increased cortical arousal, sustained activation of the HPA axis, and excessive catecholaminergic output, reinforcing difficulty initiating and maintaining sleep (39). Chronic stress and reduced vagal input promote tonic firing of the LC, which is associated with sustained wakefulness, vigilance, and cognitive hyperarousal (40). In contrast, optimal sleep–wake cycling requires a shift toward phasic LC firing and vagally mediated deactivation of hyperarousal circuits. Thus, insomnia in patients with breast cancer reflects a broader dysregulation of the central autonomic and neuroimmune networks, positioning interventions that recalibrate autonomic balance, suppress excessive arousal, and modulate inflammatory signaling, such as tVNS, as mechanistically relevant therapeutic strategies (**Figure 3**).

### Anxiety and depression: neuroimmune–HPA imbalance and vagal withdrawal

Patients with breast cancer face an elevated risk of anxiety and depression compared to both the general population and individuals with other types of cancer (125–127). Prevalence estimates vary, with studies reporting depressive symptoms in 20%–30% of patients (128). A large-scale study of 59,340 women revealed that breast cancer survivors had a 39% increased risk of depression compared to healthy controls (129). Globally, the prevalence of depression in patients with breast cancer has been reported to be as high as 30.2% (130). Anxiety prevalence is similar with one study reporting that approximately 32% of patients with breast cancer were diagnosed with anxiety (127). In a more recent study that included 283 patients with breast cancer, depression, anxiety, and stress prevalence were high (46.6%, 56.9%, and 51.9%, respectively) (131).

Depression in patients with breast cancer has been linked to poorer clinical outcomes, including lower QoL, reduced adherence to treatment, and increased risks of recurrence and mortality (132–134). A prospective study of 578 women with early-stage breast cancer reported that depressive symptoms, particularly hopelessness and high scores on the Hospital Anxiety and Depression (HAD) scale, significantly predicted reduced 5-year survival (135). In support, more recent studies associated pre-diagnosed depression with a 26% higher risk of death and post-diagnosed depression with a 50% higher risk of death (136).

Depression and anxiety in breast cancer survivors can emerge through several etiological factors, including psychological stressors, physiological dysfunction, and treatment side effects and is strongly correlated with autonomic imbalance and heightened inflammatory tone (5). Breast cancer can disrupt immune, endocrine, and neurological function, increasing the risk of depression through neurobiological changes such as reduced monoamine transmission, HPA axis dysregulation, impaired neuroplasticity, and chronic inflammation (137, 138). Chronic stress, perceived threat, and emotional distress trigger sustained activation of the HPA axis, resulting in elevated CRH signaling, and catecholamine levels that promote neuroinflammation and impair negative feedback regulation (81, 139). Reduced vagal tone limits capacity for emotional regulation, impairing adaptive engagement of prefrontal–limbic circuits that normally constrain stress reactivity and anxiety-driven hypervigilance (140, 141). Consistent with this model, lower HRV is associated with diminished emotion regulation capacity, increased depressive symptoms, and decreased stress resilience (39, 91).

Chronic sympathetic activation and dysregulated LC-NE signaling worsen anxiety symptoms, while HPA axis dysregulation maintains elevated cortisol levels that contribute to mood destabilization and glucocorticoid resistance (81). Concurrently, vagal withdrawal and heightened sympathetic tone reduce inhibitory control over inflammatory pathways, leading to increased concentrations of pro-inflammatory cytokines such as IL-6, TNF-α, and CRP, which have been strongly implicated in the development and persistence of anxiety and depressive symptoms (142, 143). Elevated cytokines can also alter monoaminergic signaling, particularly within the LC-NE system and serotonergic circuits and further increase depressive phenotypes via neuroimmune mechanisms (144). Thus, anxiety and depression in breast cancer survivors reflect a pathophysiological state characterized by HPA axis dysregulation, increased inflammation, sympathetic dominance, and impaired emotional regulation circuitry, reinforcing one another through chronic neuroimmune feedback loops (**Figure 2**, **Table 1**).

### Pain and nociceptive amplification: cytokine sensitization and autonomic dysregulation

Pain is a frequent and often persistent symptom among breast cancer survivors, arising from a combination of treatment-related tissue damage, central sensitization, and immune-driven nociceptive amplification (71). Inflammatory cytokines such as IL-1β, IL-6, and TNF-α contribute to both peripheral and central sensitization by increasing nociceptor excitability, facilitating spinal dorsal horn hyperactivity, and altering descending pain modulation pathways (81, 145). Persistent inflammation may shift pain from an acute tissue-damage response to a sustained neuromodulatory dysfunction wherein sensitized pain pathways remain hyperreactive even in the absence of peripheral input (146).

Autonomic dysregulation can exacerbate pain, particularly neuropathic pain, in cancer survivors. Increased sympathetic tone enhances adrenergic receptor-mediated sensitization of nociceptors and lowers pain thresholds in response to sensory stimuli (147). Conversely, impaired vagal tone reduces inhibitory descending pain modulation via brainstem pain pathways, including the NTS, decreasing engagement of endogenous anti-nociceptive mechanisms (91). Indeed, reduced HRV is associated with heightened pain intensity and pain-related distress in cancer survivors, suggesting that parasympathetic withdrawal contributes to dysregulated nociception (91, 147–150). Collectively, pain in patients with breast cancer reflects a convergence of cytokine-driven nociceptive sensitization and autonomic imbalance that fails to adequately recruit anti-nociceptive and anti-inflammatory control systems. This autonomic–inflammatory imbalance contributes not only to pain chronicity but also to co-occurrence with fatigue, anxiety, sleep disturbance, and depressive symptoms.

### Cancer-related fatigue: neuroimmune exhaustion and blunted vagal signaling

Cancer-related fatigue (CRF), defined by the National Comprehensive Cancer Network as a distressing, persistent, and functionally impairing sense of physical, emotional, or cognitive exhaustion disproportionate to activity (151), affects 60%–90% of patients with breast cancer and survivors across treatment phases (84). CRF is linked to poorer QoL and may be a predicter of shorter survival in patients with breast cancer (89). Rather than a passive consequence of treatment burden, CRF is now recognized as a multidimensional exhaustion state driven by dysregulated psycho-neuroimmune and metabolic systems (**Figure 3**).

One proposed mechanism underlying CRF in patients with breast cancer is elevated inflammation driven by autonomic dysregulation (67, 72, 73). Indeed, studies have shown an association between elevated inflammatory markers and fatigue (152). Elevated proinflammatory cytokines such as IL-6 and TNF-α are correlated with greater fatigue severity (72), while breast cancer survivorship studies demonstrate persistent CRF in individuals with autonomic imbalance, sleep–wake disruption, and elevated inflammatory biomarkers (145). Reduced vagal tone and a shift toward sympathetic dominance exacerbate CRF by disrupting the CAIP reflex, leading to insufficient α7 nicotinic acetylcholine receptor-mediated suppression of pro-inflammatory cytokines, thereby sustaining a state of neuroimmune activation and central fatigue signaling (35, 86). Autonomic imbalance further perpetuates CRF by weakening vagally mediated anti-inflammatory control and promoting sympathetic overdrive. In breast cancer survivors, lower high-frequency HRV has been directly associated with greater fatigue severity and elevated inflammatory markers such as IL-6, supporting the model that autonomic imbalance facilitates cytokine-driven neuroimmune fatigue loops (35, 89). Flattened diurnal cortisol rhythms and glucocorticoid receptor resistance contribute to dysregulated energy homeostasis and heightened fatigue perception (72).

### Mechanistic overlap across clusters

The psychophysiological clusters observed in patients with breast cancer share overlapping inflammatory and autonomic mechanisms that create self-reinforcing cycles of distress. Across symptom domains, we synthesize that elevated cytokines disrupt sleep architecture, promote depressive mood, increase nociceptive sensitivity, and heighten fatigue, while poor sleep, pain, and affective symptoms further increase inflammatory output. Sympathetic overactivation contributes to hyperarousal in insomnia, anxiety-driven vigilance, enhanced pain perception, and increased metabolic demand, while vagal withdrawal impairs emotional regulation and reduces activation of the CAIP. Chronic distress and low parasympathetic tone also promote tonic LC-NE overactivation, amplifying hyperarousal, disrupting sleep/wake cycles, and fostering physical and mental fatigue (40).

These converging mechanisms form interdependent psychological neuroimmune loops in which insomnia increases inflammation and emotional dysregulation, fatigue reduces activity levels and increases depressive symptoms, and anxiety heightens stress reactivity and autonomic dysfunction [**Figure 3**, (67, 98)]. The persistence and mutual reinforcement of these physiological symptoms and what underlies it may help explain why many standard single-symptom treatments produce only transient relief (2, 64). Therefore, interventions capable of recalibrating autonomic balance, dampening inflammatory signaling, and modulating arousal may offer a pathway for simultaneous alleviation of multiple symptom domains (**Figure 3**, **Table 2**).

**Table 2:** Proposed advantages of transcutaneous vagus nerve stimulation compared to standard-of-care treatments of symptom clusters in breast cancer.

Standard-of-care (SOC)	SOC limitations	Proposed advantages of tVNS
Pharmaceutical interventions (e.g., benzodiazepines, opioids, and antidepressants)	Associated with risks of severe adverse effects, drug interactions, and drug abuse or dependence.	Is a non-pharmacologic adjunct that has demonstrated safety and tolerability, with common adverse events being only mild and transient (e.g., skin irritation and headache).
Cognitive behavioral therapy (CBT)	Access is limited and underutilized. Patients prematurely drop from therapy.	Is safe, well tolerated, and feasible for repeated use in outpatient and home settings, making it suitable for long-term integration into survivorship care.
Single-symptom treatments	Often produces only transient relief because breast cancer symptoms are sustained by interconnected neuroimmune feedback loops.	Is a circuit-level intervention uniquely positioned to target multiple symptoms (clusters) simultaneously through up- and downstream stream modulation of autonomic balance and anti-inflammatory pathways.
Implanted vagus nerve stimulation (VNS)	Requires surgically invasive procedures that present added stress and immune challenges. Can produce off-target side effects (e.g., sleep apnea and dysphonia).	Is non-invasive and offers accessibility and ease of use, positioning it as a potentially viable first-line intervention compared to surgically implanted VNS devices.

Given that autonomic and inflammatory dysregulation collectively shape the emergence and maintenance of these symptom clusters in patients with breast cancer, the vagus nerve, which exerts regulatory influence over LC-NE activity, arousal, emotional control circuits, and the CAIP, emerges as a compelling therapeutic target for psychophysiological symptom cluster relief.

## Transcutaneous vagus nerve stimulation

tVNS has emerged as a promising non-invasive neuromodulatory approach capable of modulating ANS activity, inflammatory and immune signaling, cardiovascular activity, digestion, and brain networks involved in cognition, arousal, and mood (48, 57, 104, 106, 153–155). Given some potential advantages over other treatment methods (**Table 2**), tVNS has gained increasing clinical interest over the last decade. The taVNS approach is grounded in anatomical evidence demonstrating that the ABVN innervates specific regions of the external ear, particularly the tragus, cymba concha, concha, antihelix, and external acoustic meatus (156). Stimulation at these sites is theorized to elicit therapeutic effects comparable to those achieved through implanted VNS (51, 157, 158). Likewise, tcVNS targets the cervical trunk of the vagus nerve at the neck using surface electrodes placed over the sternocleidomastoid region, though its deeper anatomical location may result in less selective activation of vagal fibers (51, 60, 159). Both approaches have been shown to modulate key neuroimmune and autonomic pathways underlying symptom clusters across the cancer trajectory.

### Central pathway of VNS through the NTS

Although taVNS and tcVNS differ in their anatomical stimulation sites, both modalities primarily recruit vagal afferent pathways and converge on central mechanisms that are characteristic to vagus nerve activation (**Figure 1A**). Pivotal c-fos mapping studies provided some of the earliest neuroanatomical evidence identifying the central structures engaged by VNS. C-fos immunostaining studies in rats using stimulation parameters comparable to those used clinically for epilepsy (500 μs, 30 Hz, 1 mA, 30 s on/5 min off for 3 h) demonstrated widespread activation of vagal afferent-related regions, including the NTS, LC, cochlear nucleus, posterior amygdaloid nucleus, cingulate and retrosplenial cortices, hypothalamic nuclei, and the habenular nucleus (160). Subsequently, functional neuroimaging and electrophysiological studies confirmed overlapping activation of the NTS, LC, amygdala, insula, and prefrontal cortex, indicating that VNS primarily modulates autonomic–limbic–arousal control circuits (58, 60, 105). VNS primarily activates afferent fibers that project to the NTS in the medulla, a key autonomic integration center (60, 106). From the NTS, ascending projections innervate key neuromodulatory centers, including the LC, parabrachial nucleus, dorsal raphe, amygdala, hypothalamus, and PFC, modulating arousal, affective regulation, autonomic output, and inflammatory tone (40, 41, 161). Through these circuits, tVNS can influence several psychophysiological domains central to breast cancer-related symptom clusters.

One of the principal neurophysiological systems influenced by VNS is the LC-NE system. Historically, the LC-NE system has been implicated in arousal, but more advanced observations suggest that this system is involved in regulating a broader range of brain functions and processes including autonomic activity, attention, memory, and sensory processing (162). LC exhibits two essential types of neural discharges (tonic and phasic) that modulate cognition and behavior in response to sensory or environmental cues. Based on experimental observations in monkeys, neurons in the LC have been shown to undergo shifts in response to sensory discrimination and cognitive focus. Neurons of the LC tonically fire at high frequencies when tasks demand focused attention or during hyperarousal. Likewise, LC neurons tonically fire at low frequencies when engagement levels are low. In addition, transient firing burst preceded behavioral shifts by activating the release of NE across the brain, demonstrating LC activity support in arousal, attention, and behavioral flexibility (40, 161, 163).

The well-characterized firing patterns of the LC and its regulation of NE release are closely linked to autonomic regulation and vagal tone. LC elicits NE and acetylcholine release that act to sharpen neural signal-to-noise ratio to boost alertness to meaningful stimuli and increase arousal and emotional resilience, while concurrently activating vagal pathways that clamp overactive sympathetic arousal (164). Indeed, VNS has gained attention for its ability to safely modulate ANS activity, inflammation, neuroplasticity, attention, mood, and arousal (54, 57, 106, 154). In states of chronic stress, fatigue, and hyperarousal, the LC shifts toward high tonic firing, which promotes anxiety, insomnia, cognitive impairment, and dysfunctional allostatic stress responses (40). Disrupting high-frequency tonic firing in the LC enables the transition from high-frequency tonic LC activity to low-frequency tonic LC activity, boosting phasic LC-NE activity, a pattern linked to improved alertness, mood regulation, and sleep–wake stability (40, 161, 163, 164). VNS has been shown to modulate autonomic balance by attenuating LC tonic hyperactivity, an established driver of insomnia, hyperarousal, anxiety, and fatigue, while restoring more adaptive phasic responsiveness, contributing to improved attentional control and sleep initiation (54, 161). Through this mechanism, tVNS may offer an alternative to standard therapies by counteracting LC-driven hyperarousal that underlies persistent insomnia, anxiety, and emotional distress frequently reported in patients with breast cancer (**Table 2**).

In early studies, vagal afferent stimulation induces EEG synchronization via NTS-LC pathways, reflecting a shift toward lower tonic LC firing and increased alpha-theta activity (165, 166). Increased alpha–theta activity is linked to a shift toward parasympathetic nervous system dominance, indicating a relaxed and calm state (167). Moreover, high-frequency (tens of kHz) transcutaneous trigeminal and vagal stimulation attenuates sympathetic reactivity by modulating noradrenergic pathways, as demonstrated by reduced salivary α-amylase (an NE biomarker), suppressed galvanic skin conductance, increased skin temperature via sudomotor relaxation and vasodilation, and decreased subjective stress during shock-induced fear conditioning in healthy adults (153). Following this initial dampening of sympathetic tone, a shift toward parasympathetic dominance emerges, a sequence supported by pupil dilation studies demonstrating reduced arousal-related pupillary responses (153, 155). More investigations over the last decade have shown that both tcVNS and taVNS can reduce the sympathetic nervous system activity, as well as the psychological and neurophysiological symptoms of stress (168–172).

**Figure 4:**
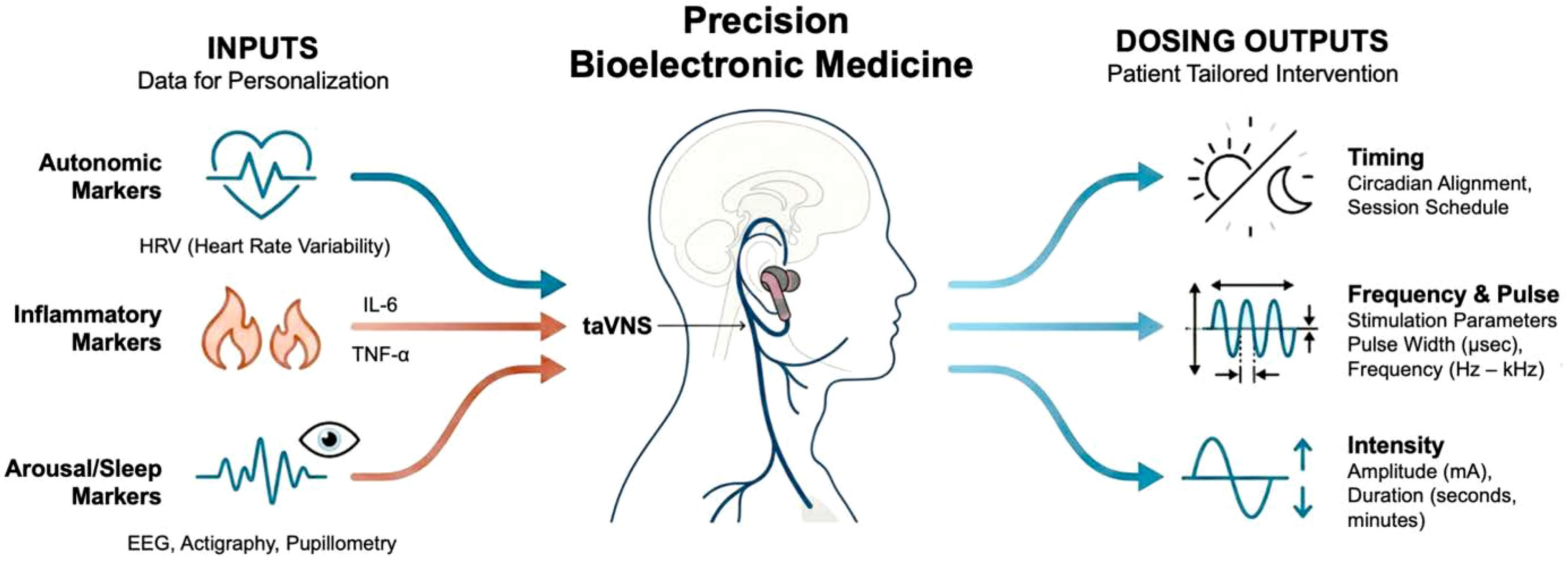
Precision-guided bioelectronic medicine for personalization of transcutaneous vagus nerve stimulation treatments in breast cancer survivorship. The schematic illustrates an approach to personalizing tVNS. As illustrated, patients may undergo phenotyping through autonomic markers (e.g., HRV), inflammatory cytokine profiles, arousal/sleep indices (e.g., EEG, pupillometry, and psychomotor vigilance reactivity), and symptom burden classification. These input data (left) can support phenotype-based categorization (e.g., hyperarousal insomnia, inflammatory fatigue, and cognitive effort-driven), guiding individualized tVNS dosing (right) strategies with respect to frequency, pulse parameters, timing (e.g., daytime vs. evening), and potential closed-loop adaptivity. Longitudinal monitoring can enable iterative recalibration to optimize therapeutic engagement and symptom outcomes. HRV, heart rate variability; taVNS, transcutaneous auricular vagus nerve stimulation; mA, milliamp; Hz, Hertz; kHz, kilohertz; EEG, electroencephalography; μsec, microsecond; IL-6, interleukin 6; TNF-α, tumor necrosis factor-alpha.

HRV is a well-established marker of autonomic function, reflecting the dynamic balance between sympathetic and parasympathetic nervous system activity (141, 173, 174). Numerous studies have demonstrated that higher HRV is associated with reduced sympathetic arousal, increased vagal tone, and greater physiological resilience to stress (59). VNS has been shown to improve HRV, reflecting enhanced parasympathetic control and better overall autonomic regulation (59, 175). It is also worth noting that high vagal activity, as indexed by elevated HRV, has been associated with improved prognosis and increased survival across multiple cancer types, including breast cancer, due to vagal-mediated decrease inflammation (50). Given that HRV is responsive to vagal stimulation and since it plays a predictive role in general health outcomes, we propose that it can be utilized as a key biomarker to advanced personalized tVNS approaches in breast cancer (**Table 3**, **Figure 4**).

**Table 3:** Components of a precision tVNS approach to the treatment of individualized symptom clusters in breast cancer.

Component	Biomarker-informed framework	Rationale
Patient stratification (phenotyping)	Patients are categorized into distinct subgroups based on measurable biological and psychological data prior to intervention (phenotyping).	Symptom clusters reflect shared underlying autonomic and inflammatory dysregulation. Stratification addresses the heterogeneity of psychophysiological and quality of life burdens.
Biomarkers used for stratification	Autonomic markers: Heart rate variability (HRV), inflammatory cytokines: IL-6, TNF-α, C-reactive protein (CRP), arousal/sleep indices: EEG, pupillometry, psychomotor vigilance, reactivity, actigraphy, and cortisol slope.	Low HRV (vagal withdrawal) identifies high-yield target groups for tVNS, particularly those with hyperarousal-related insomnia and anxiety. Elevated cytokines identify a “high-cytokine phenotype” potentially needing CAIP-engaging protocols to suppress chronic inflammatory signaling. Arousal markers act as scalable proxies of tonic vs. phasic locus coeruleus (LC) firing.
Phenotype-based targeting	Patients are matched to intervention strategies targeting their dominant drivers. For example: Hyperarousal-insomnia phenotype (characterized by reduced HRV and tonic LC overactivation); inflammatory fatigue phenotype (characterized by elevated IL-6 and CRP, as well as brain fog).	This approach addresses breast cancer distress as a cluster-based syndrome (e.g., insomnia–fatigue–anxiety constellations) rather than disconnected complaints, moving toward cluster-stratified deployment.
Dosing strategy (personalization/adaptivity)	Personalized tVNS delivery is implemented through individualized stimulation parameters in an open- or closed-loop manner.	This framework aims to optimize therapeutic engagement and adoption.
Parameters to individualize	Modality: taVNS vs. tcVNS. Frequency, pulse parameters, and duration. Timing: e.g., morning vs. daytime vs. pre-sleep administration. Intensity: Must be administered between perceptual and pain thresholds to avoid sympathetic arousal.	Dose–response studies are essential for defining optimal parameters for specific symptom constellations. Closed-loop systems dynamically adjust parameters based on real-time physiological sensing (e.g., HRV, sleep state transitions, and pupillometry) to align with patient-specific psychophysiological states.
Clinical endpoints (symptom outcomes)	Validated measures assessing the entire symptom cluster (not isolated domains).	Symptom domains include sleep disturbance, fatigue (cancer-related fatigue), anxiety, depressive symptoms, pain interference, and cognitive function.
Mechanistic endpoints (biological validation)	Parallel assessment of biological markers post-intervention to confirm mechanism engagement.	Mechanistic endpoints include changes in HRV (vagal tone restoration); reduction in IL-6, TNF-α, and CRP (CAIP engagement and cytokine suppression); and normalization of cortisol slope (HPA axis recalibration).

Beyond its modulation of LC-NE tone, VNS engages broader limbic and autonomic networks that contribute to affective and stress regulation. Afferent input to the NTS activates the dorsal raphe and enhances 5-HT signaling, while LC projections to the amygdala and hypothalamus recalibrate emotional responsiveness and arousal intensity (176, 177). tVNS further strengthens prefrontal–amygdala connectivity, facilitating top-down emotional control (178), and modulates HPA activity by dampening corticotropin-releasing hormone release and normalizing cortisol rhythms (142, 179). These central effects are complemented by enhanced cortical–autonomic coupling and increased HRV, supporting greater physiological flexibility under stress (147). In addition, VNS has been shown to reduce HPA axis excitability, a key stress pathway implicated in chronic depression (57), while also enhancing amygdala–dorsolateral prefrontal cortex connectivity, which supports improved emotional regulation (154). The HPA axis governs the body’s stress response via cortisol secretion, exerting immunosuppressive effects (180). Evidence in mice and human models demonstrate that the VNS can downregulate HPA axis hyperactivity and elicit anti-inflammatory effects via the NTS and the paraventricular nucleus (PVN) (181–184). Additionally, animal studies indicate that VNS may enhance neuroplasticity and alter neuronal firing patterns, further contributing to its antidepressant potential (185).

### Cholinergic anti-inflammatory pathway and cytokine suppression

In parallel with its central neuromodulatory effects, VNS also recruits descending parasympathetic efferent projections arising from the nucleus ambiguus and dorsal motor nucleus of the vagus, which increase vagal tone, suppress sympathetic dominance, and enhance high-frequency heart rate variability (HF-HRV), a marker of autonomic restoration (59). A cornerstone of this influence is engagement of the CAIP, a vagally mediated inflammatory reflex arc in which efferent vagus nerve activation stimulates acetylcholine release via splenic nerve interactions, thereby activating α7 nicotinic acetylcholine receptors on macrophages and monocytes and suppressing systemic inflammation through reduced production of pro-inflammatory cytokines such as TNF-α, IL-1β, and IL-6 (42, 86, 143). Under conditions of chronic inflammation, elevated CRP levels can induce a feed-forward loop that drives sustained tonic LC activity, contributing to hyperarousal, impaired attentional filtering, and sleep disruption, hallmarks of disorders such as PTSD and major depressive disorder (186, 187).

Both invasive and transcutaneous VNS have demonstrated its ability to suppress systematic inflammation and reduce circulating levels of pro-inflammatory cytokines in clinical and preclinical models of systemic inflammation (188–195), accompanied by improvements in systemic immune tone, fatigue, nociceptive sensitization, and sickness-related behavior (42, 50, 59). Given that inflammatory cytokines are mechanistically implicated in sleep fragmentation, pain amplification, cognitive slowing, negative affect, and CRF, CAIP activation represents a critical pathway through which tVNS may disrupt symptom clustering in breast cancer by simultaneously restoring parasympathetic dominance and suppressing chronic inflammatory signaling (80, 81). Although the anti-inflammatory potential of VNS has not been systematically studied in breast cancer populations, its efficacy in modulating immune function in other clinical contexts warrants further investigation to address cancer-related inflammation and its downstream effects on sleep, fatigue, and QoL in patients with breast cancer. Furthermore, discrete and continuous monitoring of cytokine levels should be included as key biomarkers in efforts to personalize tVNS therapies (**Table 3**, **Figure 4**).

### Stimulation parameters, comfort, and feasibility in survivors

Both taVNS and tcVNS have been shown to be safe, well tolerated, and feasible for repeated use in outpatient and home settings, making them suitable candidates for long-term integration into breast cancer survivorship care (51, 52, 62). These devices typically use electrical currents through surface electrodes, with customizable intensities based on sensory thresholds. Although taVNS is still in its infancy, literature illustrating safety considerations and parameters for clinical use suggest a consensus that current intensity should be administered between perceptual and pain thresholds to control for nociceptive discomfort that can lead to confounding effects (104, 196). Neuroanatomical evidence of mixed innervation in the ear and physiological studies show that painful or unconformable stimulation can recruit non-vagal nociceptive fibers associated with sympathetic arousal, particularly Aδ and C fibers from the auriculotemporal nerve or the great auricular nerve (156, 197–199). Additionally, pain or discomfort are known to decrease vagal activity and is a distracting confound that can cause emotional distress during use, potentially affecting sensory processing and influence the efficacy of taVNS (147, 149, 177). Direct evidence for tVNS-induced pain on efficacy is limited, but strong anatomical and physiological rationales suggest that it is critical that tVNS does not induce discomfort or pain as it may cause significant compounds, especially in enhancing brain plasticity, modulating arousal, and optimizing human performance (177).

Comfort and adherence consider factors such as skin sensitivity, electrical sensation tolerance, device portability, and ease of application. A systematic review of 51 studies (total *N* = 1,322) found that tVNS was safe and well tolerated in humans at the doses tested. The most common adverse events were mild and transient (e.g., skin irritation ~18%, headache ~3.6%) and no serious adverse events were definitively attributed to the intervention (49). Cancer survivors may particularly benefit from tVNS due to its minimal invasiveness, lower discomfort levels, and suitability for fatigue or insomnia-focused interventions. Personalized titration based on baseline HRV, inflammatory burden, or predominant symptom cluster may enhance response, although responder phenotyping remains an evolving area of investigation (105).

The clinical and physiological effectiveness of tVNS is highly dependent on several factors including some key stimulus parameters: frequency, intensity, pulse width, and duty cycle. These parameters can vary widely across research and clinical applications. Stimulus intensity in taVNS typically ranges from 0.1 to 50 mA, though most studies use intensities below 6 mA (200, 201). Because individual skin impedance and sensitivity differ, different dose titration methods are used in studies. These include methods for setting the intensity at individual perceptual thresholds (the lowest level at which a sensation is detected), just at the comfort thresholds (strong but comfortable), or at a fixed multiple of the threshold (e.g., 200% of perceptual threshold) (200–202). The type of electrode interface utilized includes titanium or steel ball electrodes, Ag/AgCl disks, and conductive silicones affixed to the external ear or hydrogel earbud systems designed to interface with the walls of the external acoustic meatus (106, 199). Because of electromechanical and human factors and variables discussed elsewhere, these interfaces will differentially affect patient comfort and adoptability (106).

Stimulation frequency is another critical factor, shaping the temporal pattern of neural firing and influencing clinical outcomes. Frequencies from 3 to 80 Hz are most common in clinical practice. For example, 1–5 Hz is used for migraine and gastrointestinal disorders, while 10–25 Hz is standard for conditions like major depressive disorder, anxiety, epilepsy, and insomnia. Higher frequencies, such as 30 Hz, are applied for cardiovascular regulation and Parkinson’s disease. Medium to high frequencies (100–900 Hz) have shown benefits for chronic pain, insomnia, and cognitive performance (54, 177, 203–205). Very high frequencies (1,000–20,000 Hz) are used in sub-perceptual protocols to reduce sensation while still achieving therapeutic effects, such as in rheumatoid arthritis and peripartum depression (206–208). Neuroimaging studies indicate that these high frequencies up to 20 kHz can induce lasting changes in brain connectivity without the user perceiving any electrical sensation (207). Sub-perceptual dosing, often at high frequencies > 300 Hz set at 75%–80% of the perceptual threshold, can be used to ensure participants feel no sensation, which is particularly useful for blinding in clinical trials (106).

While most taVNS protocols favor left-ear stimulation for historical reasons, evidence indicates that right-sided or bilateral stimulation can be safely implemented to enhance or differentially modulate outcomes (177, 209–212). Technical implementation of tcVNS frequently utilizes handheld devices that deliver a waveform consisting of five 5-kHz sine wave bursts, each lasting 1 ms, which repeat at a rate of 25 Hz (213). Unlike taVNS where lower intensities are required to achieve efficacy, tcVNS necessitates much higher current dosages to penetrate the skin and reach the deep-seated cervical trunk, with peak output currents reaching up to 60 mA. Based on recent modeling and empirical observations, the general tcVNS approach has been revealed to involve a high degree on nonspecific nerve and muscle stimulation that can cause off-target effects (214). Thus, we recommend that investigators and clinicians carefully and critically evaluate tVNS methods and parameters available to select approaches that safely meet the needs of their desired study or intervention objectives. Furthermore, future research should focus on how different parameters like stimulus frequency, duration, and intensity affect specific outcomes like inflammatory responses. These observations will help advance both open- and closed-loop approaches to personalizing tVNS therapies (**Figure 4**).

## Effects of transcutaneous vagus nerve stimulation on symptom cluster outcomes across clinical indications

### Inflammation

There has been a recent surge in the use of tVNS for immunomodulation across several chronic inflammatory and stress-related conditions. In autoimmune disorders like rheumatoid arthritis, tcVNS has been shown to significantly reduce CRP and interferon-gamma (IFN-γ) levels in patients with high disease activity (215). Similarly, in patients with psoriatic arthritis, tcVNS produced a 20% reduction in CRP, while those with ankylosing spondylitis exhibited decreases in IFN-γ, IL-8, and IL-10 (216). Beyond musculoskeletal conditions, taVNS has been demonstrated to reduce proinflammatory cytokines in constipation-predominant irritable bowel syndrome (217) and promote an anti-inflammatory monocyte phenotype in patients with metabolic syndrome (218). Furthermore, tcVNS effectively blocks the stress-induced activation of IL-6 and IFN-γ in patients with PTSD (213), while taVNS inhibits mental stress-induced cortisol release, suggesting a potent inhibitory effect on the HPA axis (182).

In acute neurovascular and systemic inflammatory states, taVNS serves as a non-invasive method for mitigating deleterious immune responses. Following subarachnoid hemorrhage, taVNS has been found to significantly reduce TNF-α and IL-6 in both plasma and cerebrospinal fluid, which correlates with reduced radiographic vasospasm and improved clinical outcomes (219). In the context of acute ischemic stroke involving large vessel occlusion, taVNS significantly lowered IL-6 levels, with additional reductions noted in IL-1β and IL-17α (220). The modality has also been applied to critical care and systemic infections, where it reduced cytokine production in sepsis (188) and improved inflammatory markers in patients with COVID-19 (221). Following lung lobectomy, taVNS has been shown to significantly decrease serum concentrations of CRP and IL-6 while elevating the anti-inflammatory cytokine IL-10 on the first postoperative day (189). Even in healthy volunteers, taVNS has demonstrated systemic efficacy by attenuating the whole blood transcriptomic inflammatory response to a lipopolysaccharide (endotoxin) challenge, underscoring its potential as a scalable approach for immunomodulation (211). Collectively, these findings warrant future investigations examining how tVNS affects immune responses to breast cancer and cancer therapies.

### Insomnia

Data from several randomized, sham-controlled studies show that tVNS is useful for improving chronic insomnia. In a multicenter randomized controlled trial (RCT) (*N* = 72), Zhang et al. (210) reported clinically meaningful reductions in sleep quality scores after 8 weeks of taVNS, with benefits sustained throughout the 20-week study period compared to sham. A more recent double-blind RCT (*N* = 40) likewise found that sleep quality scores improved significantly in chronic insomnia disorder after 6 weeks of taVNS, along with a significant increase in QoL (222). We recently conducted a pilot study exploring the effects of nightly taVNS on insomnia in patients with breast cancer (*N* = 20). We found that 2 weeks of bilateral taVNS targeting the external acoustic meatus with hydrogel earbud electrodes, used each night for 15 min prior to bedtime, significantly reduced insomnia index scores, improved sleep quality, decreased sleep onset latency, and enhanced sleep efficiency as observed through biometrics and patient-reported outcomes (61). In addition, we observed significant reductions in the number of nightly awakenings, CRF, and depression, while increasing HRV (61). Future tVNS development efforts should use data acquired through biometric devices in precision medicine embodiments to individualize treatment plans and programs based on sleep quality, activity patterns, and HRV (**Table 3**, **Figure 4**).

### Anxiety and depression

There is growing evidence showing that tVNS can significantly improve depressive and anxiety symptoms. A 2023 systematic review and meta-analysis of 12 RCTs (*N* = 838) showed that taVNS is effective and safe for depressive disorder, with response rates comparable to antidepressants in mild to moderate cases (223). In a randomized clinical trial in patients with MDD, taVNS was directly compared to citalopram, a selective serotonin reuptake inhibitor. The results showed that taVNS was as effective as citalopram and led to higher remission rates (224). Beyond primary MDD, a double-blind, sham-controlled RCT in post-stroke depression reported significant improvements with taVNS versus sham, reinforcing antidepressant effects across etiologies (225). In another double-blind RCT, taVNS treatment was shown to reduce anxiety through modulation of autonomic and affective circuits, aligning with improved parasympathetic tone and LC-NE arousal regulation (226). In our pilot study evaluating the effects of nightly taVNS in patients with breast cancer, we found that it produced significant reductions in depressive symptoms, while increasing HRV (61). More broadly, tVNS targeting sympathetic hyperarousal is reported to reduce anxiety symptoms and depressive symptoms in stress-related disorders, including PTSD (227) and for treatment-resistant depression (196). While trials in broader populations are warranted, current evidence supports tVNS as a tolerable, non-pharmacologic adjunct for depression and anxiety.

### Pain and nociceptive disorder

The strongest human evidence base for tcVNS is in migraine and cluster headache, with multiple RCTs showing acute pain relief and/or reduced attack frequency versus sham (45). In fact, tcVNS is Food and Drug Administration (FDA) approved for migraine and cluster headaches, supported by these randomized, sham-controlled trials showing significant reductions in headache frequency, attack duration, and pain intensity (228, 229). In a multicenter RCT of chronic migraine, tcVNS significantly reduced monthly migraine days compared to sham, with high tolerability (230). Beyond headache disorders, taVNS has been shown to reduce pain sensitivity and improve functional pain outcomes in conditions such as fibromyalgia, chronic musculoskeletal pain, and neuropathic pain, with associated increases in vagal tone and decreases in pro-inflammatory cytokines (231, 232). More specifically, taVNS has been shown to significantly reduce neuropathic pain associated with radiofrequency therapy in patients with head and neck cancer (233). Similarly, another study has demonstrated that taVNS can significantly reduce pain associated with chemotherapy-induced peripheral neuropathy in patients with cancer while improving sleep and QoL (193). Collectively, these findings support tVNS as a promising neuromodulation approach for pain relief in patients with breast cancer.

### Cancer-related fatigue

Several studies show that tVNS represents a promising therapeutic approach for alleviating fatigue symptoms across multiple clinical contexts. For example, in patients with systemic lupus erythematosus, a double-blind RCT pilot study found that taVNS significantly reduced both pain and fatigue scores compared to sham stimulation, even though inflammatory marker reductions were modest (234). A double-blind RCT investigating tVNS on human cognition after sleep deprivation showed that the active group performed significantly better on arousal, multi-tasking, and reported significantly lower fatigue ratings compared to sham (235). In an RCT of 247 women with breast cancer undergoing radiotherapy, daily tVNS was reported to significantly improve CRF 1 month post-treatment, while also improving depression scores and social functioning (236). Consistent with Yin et al. (236), we found that taVNS significantly reduced CRF after 2 weeks of use nightly use (15 min each session) prior to going to sleep (61).

### VNS and breast cancer biology

Across disease domains, tVNS shows consistent benefits for insomnia and affective symptoms, with promising improvements in cognition, fatigue, and pain. These findings are promising, but remain heterogeneous, reflecting the large variety of tVNS methods and assays used across studies. There remain gaps in efficacy in cancer populations and co-measurements of symptom relief and biometric/inflammatory/autonomic markers. Cancer biology is complex, having several hundreds of different types and involving multiple body systems. Nevertheless, two crucial etiological factors in all cancers are genetic changes or instability and the immune inflammatory response, which contribute to all stages of tumorigenesis. Importantly, tumor initiation and progression are driven by three interrelated biological processes (1): oxidative stress that induces DNA damage (2); inflammatory signaling that supports apoptotic escape, angiogenesis, and metastatic potential; and (3) heightened sympathetic activation that shapes metastatic distribution and facilitates tumor proliferation (237). VNS has been shown to reduce oxidative stress and enhance antioxidant defense pathways (238), modulate innate and adaptive inflammatory signaling to help coordinate neuroimmune responses (239), profoundly inhibit inflammation (50), enhance cellular immunity (240), and promote a more effective anti-tumor immune environment (241). Therefore, tVNS may have a prognostic and protective role in cancer that is worth mentioning. The examples discussed in this section represent only a small subset of the emerging direction and promise of VNS in cancer medicine (50, 241, 242).

## Discussion

The symptom clusters described above reflect a shared neuroimmune–autonomic dysregulation that converges on vagal withdrawal, tonic LC hyperarousal, HPA axis disruption, and persistent inflammatory amplification. As discussed, insomnia, anxiety, depression, pain, cognitive dysfunction, and CRF are sustained not by isolated etiologies, but by interacting psychophysiological loops anchored in central vagal pathways. Evidence described demonstrates that tVNS exerts clinically meaningful effects across these symptom domains. Together, this mechanistic and clinical overlap suggests that tVNS is not merely a symptom-alleviating tool, but a circuit-level intervention suited to treating breast cancer distress as a cluster-based syndrome (**Table 1**, **Figure 2**) rather than as a series of disconnected complaints.

Vagal stimulation offers a uniquely upstream therapeutic approach because it interfaces with multiple systems simultaneously that jointly govern autonomic balance, affective regulation, and anti-inflammatory modulation (**Figures 1**, **3**). This aligns findings from studies showing cross-symptom improvements in sleep, mood, fatigue, and nociception following tVNS in clinical and preclinical trials. In this way, tVNS is conceptually positioned not as a niche or adjunctive therapy but as a promising bioelectronic intervention for targeting the architecture of breast cancer symptom clustering (**Table 1**). Thus, a translational tVNS model for patients with breast cancer should move beyond single-symptom applications and toward cluster-stratified deployment, wherein individuals presenting with insomnia–fatigue–anxiety constellations, pain–cognitive fog networks, or depression–hyperarousal phenotypes are matched to vagally mediated intervention strategies targeting their dominant autonomic–inflammatory drivers (**Figures 3**, **4**). This cluster-centric framing supports precision survivorship paradigms and sets the foundation for biomarker-based personalization strategies (**Figure 4**). However, to make biomarkers truly useful for guiding tVNS treatments in breast cancer, future studies should determine specific, measurable cutoff values that define different patient types. Once these quantitative thresholds are established, clinicians may use biomarker data to more confidently match patients with the most appropriate tVNS protocols.

To rigorously evaluate tVNS in breast cancer survivorship, future clinical trials should be designed to assess both symptom outcomes and mechanistic pathway engagement. Standard endpoints should include validated measures of sleep disturbance, fatigue, anxiety, depressive symptoms, pain interference, and cognitive function, ideally grouped by symptom clusters rather than isolated domains. Given the heterogeneity of psychophysiological burden across breast cancer survivorship, personalization of tVNS delivery may enhance clinical efficacy by aligning stimulation parameters with distinct autonomic and inflammatory phenotypes individualized to patients of varying or domains (**Table 3** and **Figure 4**). Additional research is required to determine how specific tVNS methods and parameters affect different symptom outcomes across individuals to develop phenotypic response profiles and fully realize a precision-guided approach.

HRV provides a non-invasive marker of vagal tone and has been consistently associated with fatigue, insomnia, mood disturbance, and broader symptom clustering (37, 92). Individuals characterized by pronounced vagal withdrawal as determined by HRV may represent a high-yield target group for tVNS, particularly when addressing hyperarousal-related insomnia, anxiety, and cognitive inefficiency. As mentioned above, however, more research is needed to clearly establish these phenotypes and response profiles. Inflammatory profiling similarly also offers a pathway for guiding tVNS selection and monitoring. Elevated IL-6, TNF-α, and CRP levels have been linked to pain sensitization, CRF, depressive symptoms, and psychomotor slowing (72, 145). Patients exhibiting a high-cytokine phenotype may respond preferentially to CAIP-engaging tVNS protocols or to combination regimens that integrate anti-inflammatory lifestyle interventions (e.g., exercise, nutrition chances, or stress-reduction therapies). Dynamic cytokine tracking may also serve as a mechanistic engagement marker, enabling assessment and real-time titration of stimulation based on inflammatory reflex responsiveness.

Neurocognitive arousal profiling may further refine subgroup identification. Digital biomarkers such as pupillary dilation, polysomnography, EEG and other imaging, psychomotor vigilance task performance, or task-related HRV reactivity provide indirect yet scalable proxies of tonic vs. phasic LC firing and cognitive effort burden. When used alongside subjective indicators such as perceived fatigue or mental load, these markers may support a precision neuromodulation classification framework and better therapeutic outcome. Parallel assessment of autonomic and inflammatory biomarkers, such as HRV, IL-6, TNF-α, CRP, cortisol slope, and pupillary reactivity, can help determine whether improvements are mediated through LC–HPA–CAIP recalibration, offering biological validation of the proposed mechanism-based survivorship model. Incorporating appropriate assessments, digital phenotyping of stress and fatigue load, and wearable HRV tracking may allow dynamic evaluation of within-person tVNS effects on diurnal regulation and reactivity to daily demands (**Table 3**, **Figure 4**).

The integration of autonomic, inflammatory, and arousal biomarkers is now being operationalized through next-generation bioelectronic medicine. Closed-loop tVNS systems leverage real-time physiological sensing (e.g., HRV, electrodermal activity, sleep state transitions, and pupillometry) to dynamically adjust stimulation parameters in accordance with autonomic or arousal fluctuations (106). By delivering stimulation during windows of vagal receptivity or sympathetic overdrive, these feedback-responsive systems optimize frequency, duration, timing, and modality (e.g., taVNS vs. tcVNS) to align with patient-specific psychophysiological states (**Figure 4**). Indeed, dose–response studies comparing stimulation frequency, duration, and timing relative to behavioral interventions are limited and will be essential in defining optimal parameters for specific symptom constellations. This biomarker-informed, adaptive dosing framework represents a shift from uniform stimulation protocols toward precision, phenotype-aligned bioelectronic interventions that enhance mechanistic engagement and improved therapeutic outcomes. By embedding mechanistic biomarkers, cluster-specific endpoints, and psychophysiological stratification strategies, clinical trials can accelerate the development of precision neuromodulation paradigms for survivorship care (**Table 3**, **Figure 4**).

Breast cancer survivorship is frequently marked by a constellation of psychophysiological symptoms that reflect a shared axis of autonomic dysregulation, inflammatory persistence, and central arousal imbalance. Non-invasive vagus nerve stimulation offers a circuit-level intervention uniquely positioned to target these symptom clusters through modulation of LC–HPA–CAIP pathways, restoration of parasympathetic tone, and attenuation of neuroimmune amplification. Evidence from diverse clinical populations supports its efficacy across individual symptom domains, and emerging feasibility data suggest its translational relevance for breast cancer survivors. By aligning tVNS delivery with psychophysiological phenotyping and integrating it into multimodal survivorship care models, this neuromodulatory approach may enhance the effectiveness of behavioral, rehabilitative, and anti-inflammatory interventions (**Table 3**, **Figure 4**). While direct evidence in breast cancer-specific populations remains in early development, the mechanistic coherence and cross-domain efficacy of tVNS support its advancement as a promising tool in neuroimmune-informed survivorship care. tVNS holds promise not only for symptom alleviation but also for sustained autonomic resilience and immune homeostasis. As oncology continues to evolve toward biologically grounded, circuit-targeted intervention strategies, non-invasive vagal neuromodulation may play a pivotal role in shaping the future of personalized neuroimmune-based supportive cancer care.

## Data Availability

The original contributions presented in the study are included in the article/supplementary material. Further inquiries can be directed to the corresponding author.
